# Work-Recreation Balance, Health-Promoting Lifestyles and Suboptimal Health Status in Southern China: A Cross-Sectional Study

**DOI:** 10.3390/ijerph13030339

**Published:** 2016-03-19

**Authors:** Shengwei Wu, Zhengzheng Xuan, Fei Li, Wei Xiao, Xiuqiong Fu, Pingping Jiang, Jieyu Chen, Lei Xiang, Yanyan Liu, Xiaoli Nie, Ren Luo, Xiaomin Sun, Hiuyee Kwan, Xiaoshan Zhao

**Affiliations:** 1 School of Traditional Chinese Medicine, Southern Medical University, Guangzhou 510515, China; a2278197a@163.com (S.W.); 13268268154@163.com (Z.X.); leephialf@126.com (F.L.); xw76888@126.com (W.X.); 13427550499@163.com (P.J.); jieyu@smu.edu.cn (J.C.); xianglei9527@163.com (L.X.); siyecao2015@163.com (Y.L.); nx117@163.com (X.N.); luoren2014@126.com (R.L.); sunxiaomin198001@163.com (X.S.); 2 Department of Traditional Chinese Medicine, Nanfang Hospital, Southern Medical University, Guangzhou 510515, China; 3 School of Chinese Medicine, Hong Kong Baptist University, Hong Kong 999077, China; 13480405@life.hkbu.edu.hk

**Keywords:** work-recreation balance conditions, suboptimal health status (SHS), health-promoting lifestyles

## Abstract

Suboptimal health status (SHS)—an intermediate state between health and illness—refers to functional somatic symptoms that are medically undiagnosed. Although SHS has become a great challenge for global public health, very little about its etiology and mechanisms are known. Work-recreation balance is a part of work−life balance, and is related to stress which greatly influences health status. We therefore carried out a cross-sectional investigation between 2012 and 2013 within a clustered sample of 24,475 individuals aged 15−60 years from a population in southern China. In so doing, we hoped to illuminate the associations between work-recreation balance conditions, healthy lifestyles, and SHS. Work-recreation balance conditions were categorically defined by frequency (“rarely, sometimes, or always”). Health-Promoting Lifestyle Profile (HPLP-II) was used to evaluate the level of healthy lifestyles, and the medical examination report and Sub-Health Measurement Scale V1.0 (SHMS V1.0) were both used to evaluate health status. The ratio of SHS (46.3%) is higher than health status (18.4%) or disease status (35.3%). Overall, 4.9% of respondents reported the lowest level of work-recreation balance, and they scored lower on both the HPLP-II and SHMS V1.0 compared with those who frequently maintained a work-recreation balance. Significant association was found between work-recreation balance behaviors and healthy lifestyles (*p* < 0.001) after demographic adjustment. In comparison with those reporting a frequent work-recreation balance, individuals whose work-recreation balance was categorically “rare” were 1.69 times as likely to develop SHS (odds ratio (OR): 1.69, 95% confidence interval (CI): 1.49–1.92), and those with infrequent work-recreation balance (“sometimes”) were 1.71 times more likely to develop SHS (OR: 1.71, 95% CI: 1.62–1.81). These findings suggest that work-recreation balance conditions are significantly associated with, and seem to be accurate behavioral indicia of a healthy lifestyle. Poor work-recreation balance is associated with increased risk for SHS; thus, a healthier lifestyle that maintains a work-recreation balance should be promoted in order to reduce the development of SHS or disease in southern China.

## 1. Introduction

Due to the rapid development of the social economy and the accelerating rhythm of life, tasks and targets keep springing up both in work and daily life. Disruptions of work−life balance, which result in dissatisfaction, depression and intense pressure, have become a pervasive social issue. Thus, the research of work−life balance has gained increasing attention from healthcare professionals in recent years. Work−life balance is defined as individuals being able to properly prioritize activities related to their job, family, community, and self-development [1]. Work−life conflict can be regarded as a work-related stressor, which is associated with health problems [2,3]. Previous studies have provided evidence of associations between poor work−life balance and health complaints, e.g., based on self-reported health measures [4,5], as well as psychological strain and distress [6,7], depression and mental health disorders [8,9], burnout [10,11], psychosomatic symptoms including lack of appetite and fatigue [12], *etc*. The term work−life balance is often mentioned in public discussion and pursuing harmony between work and personal life has become a new policy priority within the European Union [13].

However, we often feel exhausted after juggling busy work schedules and daily chores. There is no doubt that work consumes most of our time and energy. Moreover, the range of life activities is relatively wide, including commitment to family and friends, religion, leisure, community, *etc*. Thus, the idea of work−life balance is hard to accomplish and can somehow create more stress and anxiety. It is thus important to unwind and decrease stress to prevent burnout. Among all the activities, recreation is of great importance in work−life balance, for it can arouse positive emotions, promote self-efficacy, increase competency, and act as buffers for stress according to research [14]. There is some consensus on the definition of recreation; recreation is generally understood as any activity that people participate in during their free time, and which they enjoy and recognize as having socially redeeming values. Examples of recreational activities are numerous, including activities that divert, amuse, or stimulate, such as sports, music, games, travel, and dance [15]. Recreation is necessary for lifelong wellbeing in humans, especially because it helps relieve stress [16]. Tsai *et al.* [17] found that perceived work-related stress had an indirect impact on health-related quality of life. Furthermore, Moreau *et al.* [18] pointed out that increasing the level of leisure activity would be expected to enhance not only self-rated health and other individual health indices, but also outcomes relevant to the functioning of organizations, such as sickness and absence. Thus, it is apparent that work-recreation balance is most likely related to both lifestyle and health status.

Currently, people have begun to pay much more attention to health as social economics develop and the pace of life increases. It has been categorized into three distinct types according to the general concept of health status; namely health, disease, and the intermediate state between health and disease, referred to as suboptimal health status (SHS). SHS refers to functional somatic syndromes or symptoms that are medically undiagnosed, such as non-specific pain (e.g., foot pain or pelvic pain), headaches, anxiety, chronic fatigue, fibromyalgia, irritable bowel syndrome *etc.* [19,20,21,22]. As a whole, SHS can be characterized by a decline in physical function, mental function and social capacity [23], which leads to impaired quality of life, frequent hospital visits, and incurrence of significant medical expenses [19].

In a previous investigation, undertaken by the authors of the current study, it was found that the prevalence of SHS in southern China [23] is approximately 56% and that SHS is also becoming a global issue [24]. According to current intervention strategies, the prevention and effective treatment of early-stage illness was highly emphasized [25,26], and prevention of SHS is a logical place to begin. However, further studies are needed, both to better understand the etiology of SHS, and to elucidate the associated mechanisms.

Although the aforementioned study has demonstrated the prevalence of SHS and its consequences, it lacks research considering the correlation between work-recreation balance and SHS—a gap filled by the present study. As poor work-recreation balance can be considered as a work-related stressor—and thus significant with respect to health status—we hypothesized that it is related to SHS. Consequently, current study aims to collect data to support the above hypothesis. As far as we know, the research on work-recreation balance with respect to SHS is lacking and there is a paucity of data in relation to the potential association between work-recreation balance and health status within the Chinese population. There is therefore an urgent need for a serious investigation to address this issue in order to prevent a lack of awareness further jeopardizing people’s health or harming their efforts to make changes to improve their work-recreation balance. Therefore, we conducted a comprehensive cross-sectional study within southern China, the spearhead of economic development with its massive workforce, in order to elucidate associations between work-recreation balance, healthy lifestyles, and the risk of SHS.

## 2. Experimental Section

### 2.1. Sampling and Procedures

This cross-sectional descriptive survey was performed on 28,144 individuals aged 12–80 years from 14 units which were selected by a three-stage, stratified, random cluster sampling. To be specific, six cities representative of economic, demographic, and distribution characteristics in southern China were selected for the study. In the first stage of sampling, one district was randomly selected from each of the selected cities. In the second stage, one community was randomly selected from each designated district. In the final stage, 14 units were selected randomly from each designated community, including schools, companies, government agencies, or factories etc. A self-administered questionnaire was allocated to each staff member working in the aforementioned 14 units by on-site distribution or mail. A total of 24,475 individuals (11,891 men and 12,584 women) aged 15−60 were included for the current analysis, which resulted in a valid response rate of 87.0%. The respondents were informed of the purpose of this study and their rights in accordance with the Declaration of Helsinki. Verbal informed consent was obtained from each respondent prior to the study. Respondents less than 18 years old lacking full civil capacity were required to provide informed consent under guardian’s authorization. All data were kept strictly confidential. The study was approved by the ethics committee of Nanfang Hospital in Guangzhou, China (2012) LunShenZi (No. 035), which also approved the consent procedure.

### 2.2. Questionnaires

The questionnaire was comprised of self-designed items and standardized questionnaire items. The self-designed items mainly focused on general demographic characteristics, covering age, gender, body mass index (BMI), marital status, level of education, occupation, smoking status, and alcohol consumption. Adults were classified as “overweight” or “non-overweight” using a body mass index of 24 kg/m^2^ as a cut-off [27]. As for students less than 18 years old, the cut-off was made by referring to the standard put forward by Group of China Obesity Task Force [28]. The exposure variable was the frequencies of work-recreation balance assessed by the question “how often do you balance your work and recreation” (since it is mental work, studying was defined as work for students), and this variable was defined, according to the frequency of the behavior, using a three-point response format: “rarely, sometimes, always” (the rating score ranged from 1 to 3). The standardized items were internationally applied scales including the “Sub-Health Measurement Scale V1.0 (SHMS V1.0,)” and the “Health-Promoting Lifestyle Profile (HPLP-II)” to provide assessment of participants’ health status and health-promoting lifestyles, respectively. Uniform instructions were furnished by trained investigators, and questionnaires were expected to be self-completed by participants in approximately 30 min.

### 2.3. Suboptimal Health Status (SHS) Assessment

The SHMS V1.0, developed by our research group, is a self-reporting scale for providing assessment on health status. This Chinese version of the scale, combined with a medical examination self-report, was adopted to evaluate the health status of our participants in this study. According to Chinese research data, the SHMS V1.0 displayed an excellent level of content reliability and validity, supported by Cronbach’s alpha and split-half reliability coefficients of 0.92 and 0.83, respectively [29]. Within this study, the Cronbach’s alpha was 0.91 for the total SHMS V1.0 and the Cronbach's alpha for three dimensions of total SHMS V1.0 ranged from 0.82−0.85. The scale was comprised of 39 items, amongst which 35 can generally be categorized into three dimensions (physiological symptoms: 14 items further distributed into four factors: physical symptoms, organ function, body movement function, and energy; psychological symptoms: 12 items further distributed into three factors: positive emotion, mental symptoms, and cognitive function; social symptoms: 9 items further distributed into three factors: social adaptation, social resources, and social support). Each item was rated on a self-evaluated five-point Likert scale, ranging from 1 (never) to 5 (always), *i.e.,* 1 = never, 2 = rarely, 3 = sometimes, 4 = usually, 5 = always. A total of 15 items were reverse arranged. Respondents were inquired to rate the frequencies item by item according to their experiences during the past month. The scores of each factor and total scores were then calculated. A lower score indicated an increased likelihood of SHS (*i.e*., poor health status). Prior to surveys being undertaken, respondents attended an annual health examination in hospital, which contained medical history, a physical examination, biochemical and blood hematology analyses, chest radiography and resting electrocardiogram (ECG). After exclusion of respondents who were diagnosed with clinical disease by medical doctors, SHS was diagnosed when any aspect of physiological, psychological, and society dimensions of SHMS V1.0 was lower than their threshold scores of 68, 67, and 67, respectively. Moreover, respondents were considered healthy after exclusion of SHS and disease state [29,30].

### 2.4. Lifestyle Assessment

The HPLP-II has been widely applied as a measuring instrument for assessing health-promoting behaviors with good reliability and validity [31,32]. In China, Wang *et al.* reported a Cronbach’s alpha of 0.93 for the total HPLP-II, 0.63−0.82 for its subscales [31]. Within this study, the Cronbach’s alpha was 0.94 for the total HPLP-II, 0.77−0.86 for its subscales. The scale consists of 52 items and contains six subscales: self-realization, health responsibility, sports and exercise, nutrition, interpersonal relationships, and stress management. Based on the self-reported four-point Likert scale (“never, sometimes, usually, always” rating from 1 to 4), the questionnaire was used to evaluate the frequency of health-promoting behaviors. Possible total scores of HLPLP-II range from 52 to 208, with a higher score indicating a higher level of health-promoting behaviors. A total score ranging from 52–103 corresponds to a poor level, 104–156 to a general level, and 157–208 to a good level. For self-realization, health responsibility, nutrition, and interpersonal relationships subscales, a score ranging from 9−17 is rated as at a poor level, 18−27 a general level, and 28−36 a good level. For the sports and exercise subscale, as well as the stress management subscale, a score ranging from 8−15 is rated as poor, 16−24 as general, and 25−32 as good.

### 2.5. Statistical Analysis

All data were analyzed using SPSS 19.0. Descriptive statistics were used to describe the overall population. Univariate analyses were used to compare variations in frequencies of work-recreation balance; for both health-promoting lifestyle and health status (including its factors) scores, one-way ANOVA and Bonferroni correction for *ad hoc* multiple comparisons were used. Based on demographic variables, multinomial logistic regression was used to estimate typical profiles for individuals with poor work-recreation balance. The reference group refers to the respondents with the lowest level of exposure, *i.e.*, those who habitually maintained work-recreation balance, or who had excellent lifestyle behavior. It was similar to analyzing the associations between work-recreation balance and health status using multinomial logistic regression. Identified by the backward stepwise method, potential confounders including age, gender, BMI, marital status, education level, occupation, unit, drinking behavior, and smoking status, were adjusted. All *p*-values were two tailed, and *p* < 0.05 was considered to be statistically significant.

## 3. Results

### 3.1. General Characteristics of Subjects

In total, 24,475 participants (11,891 men and 12,584 women) were included. Table 1 summarizes the detailed distribution of the participants’ demographic characteristics. The mean age was 27.05 years old. The percentages of “healthy”, “SHS,” and “disease” groups were 18.4% (4503), 46.3% (11,337), and 35.3% (8635), respectively. The respiratory, digestive, endocrine, and autoimmune systems were involved according to major disease reported, and included chronic rhinitis (10.7%), chronic pharyngolaryngitis (9.5%), chronic gastritis (4.6%), gynecopathia (4.3%), chronic insomnia (3.8%), hemorrhoids (3.5%), breast disease (3.1%), fatty liver (2.3%), hypertension (2,0%), chronic bronchitis (1.9%), gastroduodenal ulcer (1.9%), rheumatic disease (1.8%), hyperlipemia (1.3%), chronic joint disease (1.2%), chronic otitis media (0.9%), thyroid disease (0.9%), cholecystitis (0.7%), chronic hepatitis (0.5%), heart disease (0.5%), diabetes (0.4%), prostatic diseases (0.4%), cancer (0.4%), asthma (0.3%), cerebrovascular disease (0.2%), arteriosclerosis (0.1%), tuberculosis (0.1%), and chronic nephritis (0.1%).

### 3.2. Profile of Individuals with Poor Work-Recreation Balance

Table 2 presents baseline population demographics of the group characterized by poor work-recreation balance. Participants who reported poor work-recreation balance were more likely to be young (aged mainly between 25 and 45 years), male (significant at the lowest level of work-recreation balance frequency), married, with lower level of formal education, and higher level of smoking and drinking behaviors.

### 3.3. The Comparison of Work-recreation Balance Frequency in Health-Promoting Lifestyles and Health Status

Table 3 displays results of one-way ANOVAs to compare work-recreation balance frequency in both indicators of a health-promoting lifestyle and health status. Of the respondents, 4.9% reported the lowest level of work-recreation balance. Significant differences were found with respect to six dimensions of a health-promoting lifestyle and work-recreation balance frequency; moreover, analyses showed significant differences in the total HPLP-II score (F_2, 24475_ = 5239.31, *p* < 0.001). It was also significantly different after the Bonferroni correction for multiple comparisons (*p* < 0.001). On the contrary, individuals who habitually maintained work-recreation balance scored significantly higher on health status scales and for total SHS score (F_2, 24475_ = 2087.83, *p* < 0.001). Bonferroni *ad hoc* tests revealed significant differences compared with the work-recreation balance frequency (*p* < 0.001).

### 3.4. Work-Recreation Balance Frequency and Health-Promoting Lifestyles

Table 4 reveals the associations between work-recreation balance frequency and health-promoting lifestyles. It showed a significant association between work-recreation balance frequency and ahealth-promoting lifestyle using multivariable regression analyses with adjusted demographic variables (*p* < 0.001). Compared with habitual work-recreation balance keepers, those with the lowest work-recreation balance frequency were approximately 388 times more likely to exhibit a poor health-promoting lifestyle (odds ratio (OR) 388.02, 95% confidence interval (CI) 160.03–940.84); individuals who infrequently kept work-recreation balance were almost 132 times more likely to exhibit a poor health-promoting lifestyle (OR 132.62, 95% CI 100.41–175.16). Furthermore, rare or infrequent work-recreation balance had clear associations with other components of a healthy lifestyle, including poor stress management (OR 826.24, 95% CI 499.16–1367.66), poor interpersonal relationships (OR 125.73, 95% CI 87.75–180.15), poor self-realization (OR 36.18, 95% CI 28.87–45.35), poor nutrition (OR 23.52, 95% CI 14.03–39.44), less sport and exercise activities (OR 18.88, 95% CI 11.27–31.63), and poor health responsibility (OR 15.99, 95% CI 6.60–38.75).

### 3.5. Work-Recreation Balance Conditions and Health Status

Table 3 and Table 5 exhibited the association between work-recreation balance frequency and health status. Positive frequency responses were noted with respect to the likelihood of both SHS and disease, and corresponded with work-recreation balance frequency. Thus, respondents reporting a low frequency were 1.69 times as likely to develop SHS (OR 1.69, 95% CI 1.49–1.92), and individuals who reported infrequent work-recreation balance were 1.71 times more likely to develop SHS (OR 1.71, 95% CI 1.62–1.81), relative to individuals who habitually maintained a work-recreation balance (*p* < 0.001). Similarly, the lowest frequency group was approximately 1.9 times more likely to develop disease, compared with individuals habitually maintaining a work-recreation balance (OR 1.88, 95% CI 1.65–2.14). Significant differences were found on the likelihood of developing SHS, with respect to three symptomatic dimensions (physiological, psychological, and social); for instance, compared with individuals who habitually maintained a work-recreation balance, individuals with the lowest frequency of work-recreation balance were approximately 3.8 times more likely to exhibit physiological SHS (OR 3.78, 95% CI 3.21–4.45). Moreover, ORs of 5.24 (95% CI 4.35–6.33) and 6.77 (95% CI 5.46–8.38) were associated with physiological and social SHS, respectively, within the same group. The results shown in Table 3 remained consistent over all the analyses, and significant differences were found for each factor of the three aforementioned dimensions and work-recreation balance frequency (specifically, low scores for physical symptoms tend to relate to poor work-recreation balance).

## 4. Discussion

This is the first comprehensive survey to determine the relationship between healthy lifestyles, SHS, and work-recreation balance conditions within the Chinese population. Although work−life balance has garnered a great deal of attention from healthcare professionals, few studies have focused on work-recreation balance or its correlation with health. Our findings indicate that a poor work-recreation balance is relevant to multiple types of unhealthy lifestyles. Significant associations were found between frequency of work-recreation balance, and prevalence of SHS and disease status. In particular, a frequency-response effect was noted, with greater awareness of work-recreation balance being correlated with decreased prevalence of SHS and disease status. For behaviors pertaining to work-recreation balance, significant associations were found for three dimensions of health status (physiological, psychological, and social).

SHS is considered to be an intermediate status between disease and health, which is characterized by a decline in vitality, in physiological function and in the capacity for adaptation within a period of three months, and is regarded as a subclinical, reversible stage of chronic disease [25,26,33]. Multiple factors were found influential to SHS, including gender, age, physical activities, dietary habits, emotional problems, social adaptation, *etc.* [34,35,36]. Based on these, we developed SHMS V1.0 and adopted it as an instrument in this study. It is a multidimensional, self-reporting symptom inventory concerning physiological, psychological, and social health [29] that presents a more comprehensive interpretation of health as reflected in the WHO’s definition [37].

The prevalence of SHS in this survey is 46.3%. As predicted, significant interactions were found between SHS prevalence or disease states, and work-recreation balance frequency. Positive frequency responses were evident, both for the likelihood of developing SHS or disease, with work-recreation balance conditions (*i.e.*, poor work-recreation balance coincided with increased likelihood of an impaired health response). Kahan and colleagues found that people who displayed more symptoms such as hyperlipidemia, hypertension, obesity, osteoporosis *etc.* engaged in fewer leisure and sports activities, supporting the notion that sports activity has positive effects on health [38]. Johannesson *et al.* [39] showed that low physical activity is related to chronic fatigue, and according to a review article by Puetz [40], there is agreement among studies suggesting a strong, dose-response relationship between physical activity and reduced feelings of fatigue. In addition, previous studies have reported that frequent recreation is associated with better self-reported health and physical functioning, and psychological wellbeing [41,42,43]. The studies mentioned above have indicated that insufficient levels of vigorous recreational physical activity, due to poor work-recreation balance, increase SHS or disease risk. Moreover, our results showed a significant interaction between work-recreation balance conditions and health status, assessed with respect to three symptomatic dimensions and their factors (physiological, including factors related to physical symptoms, organ function, body movement function, and energy; psychological, including factors related to positive emotion, mental symptoms, and cognitive function; social, including social adaptation, social resources, and social support). Consistent with previous studies, several studies have shown that a poor work−life balance (recreation is considered part of life) is related to low energy [44], emotional exhaustion [11], and lack of social support (including receiving support from one’s partner, participating in recreational activities, and maintaining good relationships with colleagues) [45].

The decreased frequency in which people participate in recreational activity during leisure time when they are under stress at work may play a causative role with respect to suboptimal physical fitness, leading to SHS or disease. Participation in various types of recreational activity has been shown to be effective in buffering symptoms of depression and anxiety [46]. More specifically, participation in recreational activities (e.g., walking, cycling, yoga, music, meditation, or social groups) may help to provide relaxation [47] and promote social support [48], and culturally relevant activities have been shown to be successful in reducing depression and anxiety [49]. Furthermore, there is compelling evidence of a dose-response relationship for recreational physical activity in preventing cardiovascular disease, stroke, and hypertension [50], in addition to SHS [51]. Thus, it is essential to emphasize the need for maintaining a work-recreation balance.

In this study, a significant relationship was found between work-recreation balance conditions and health-promoting lifestyles, with consequences, in particular, for stress-management capability. For those with a better balance between work and recreation time, evidence was found for healthier overall lifestyle patterns, including effective stress-management capabilities, enhanced interpersonal relationships, improved attention toward personal nutrition, and more frequent engagement in physical activity. For instance, an individual with poor work-recreation balance may become a stressed worker, and may consequently show increased consumption of energy-rich foods that are high in saturated fats and sugars, as well as reduced physical activity leading to obesity [52,53]. In contrast, individuals presenting with a good work-recreation balance exhibited a tendency toward participation in physical activities [38]. Assessment of work-recreation balance conditions may therefore be a helpful tool for predicting a healthy lifestyle.

Accordingly, a poor work-recreation balance can be considered a part of unhealthy lifestyle, while an unhealthy lifestyle is reportedly associated with increased risks of SHS [20,54], obesity [55], chronic rhinitis [56], coronary heart disease [50], breast cancer [57] *etc.* Thus, people who fail to maintain a work-recreation balance tend to have an unhealthy lifestyle and a greater risk of suffering from physical and psychological disease, a correlation proved by our study. The findings that emerged from this study urge people to balance their work and recreation as soon as possible. In addition, employers should set reasonable break times for employees for the benefit of decreasing health insurance expenses. 

Moreover, our research analyzed the association between demographic characteristics and work-recreation balance. Our results showed that the group aged from 25 to 45 years was more representative of the lowest level of work-recreation balance, compared with the older age group. Due to substantial academic as well as work commitments at this age, young people exposed to significant levels of stress may fail to allocate their time appropriately. Previous studies have consistently demonstrated that high-strain jobs are the most stressful for workers and lead to a near absence of activities in their leisure time [58,59,60]. For females, unmarried individuals, and those with a higher level of education, significant associations were found and the results may indicate a higher likelihood of work-recreation balance; similar findings have also been previously reported [58,61,62]. Further studies that contribute to better strategies for communicating the importance of work-recreation balance are therefore required, in order to achieve the health benefits of specific population demographics. Our results remind us that maintaining a work-recreation balance improves health and wellbeing, due to the individual’s awareness of healthy lifestyle decisions. Further studies should be carried out to examine time allocation, pressure level, types (and effectiveness) of recreational activity—and to illuminate the potential mechanisms that result in SHS and disease at the individual or population level—via larger and longer trials.

### Strengths and Limitations

As far as we know, this is the first comprehensive survey to demonstrate the associations between work-recreation balance conditions and SHS within the Chinese population. Additionally, our findings reveal the results that work-recreation balance behaviors are significantly associated with physiological, psychological, social health.

Several limitations to the study should be addressed as well. Work-recreation balance was self-evaluated with subjectivity and with only one item, which measured how often individuals balance their work and recreation. Consequently, it was not possible to learn about different dimensions of work-recreation balance. As is the case with any other questionnaire survey, there may exist some inherent inaccuracy or bias from the respondents. Moreover, it may not accurately represent the population because of a disproportionate number of students in this survey. Furthermore, though several potential factors related to health and lifestyle were taken into account, data regarding different dimensions associated with work-recreation balance were not collected and analyzed (e.g., time allocation, pressure level, types and quality of recreational activity). Thus, studies should aim at correcting these limitations in the future. Additionally, the study’s cross-sectional design can only describe statistical relationships between work-recreation balance and SHS. The next step is to therefore design a prospective study with the aforementioned problems addressed.

## 5. Conclusions

In conclusion, the current cross-sectional survey—the first to assess work-recreation balance conditions in relation to healthy lifestyles and SHS within the Chinese population—revealed a number of significant associations. Strong correlations between work-recreation balance behaviors and SHS, as well as physiological, psychological, and social health, were demonstrated. In view of these findings, healthier lifestyles that maintain a work-recreation balance should be promoted, thereby contributing to prevention of SHS or disease and ultimately benefiting the social economy.

## Figures and Tables

**Table 1:** General characteristics of subjects (*n* = 24,475).

Characteristic	n	%
Age		
≤25	13,909	56.8
>25 and ≤35	5284	21.6
>35 and ≤45	3900	15.9
>45	1382	5.6
Gender		
Man	11,891	48.6
Woman	12,584	51.4
Body mass index (BMI)		
Non-overweight	15,454	63.1
Overweight	9021	36.9
Marital status		
Unmarried	14,510	59.3
Married or others	9965	40.7
Education level		
Less than junior high school	2460	10.1
High school or college	9135	37.3
Bachelor degree or above	12,880	52.6
Professional		
Professional	5384	22.0
Manager	3649	14.9
Clerk	239	1.0
Soldier	88	0.4
High school student	10,087	41.2
Production personnel	128	0.5
Operating personnel	2802	11.4
Business, service personnel	483	2.0
Freelancer	860	3.5
No occupation (housewife, *etc.*)	51	0.2
Others	704	2.9
Smoking status		
Never	20,804	85.0
Yes	3395	13.9
Previously	276	1.1
Drinking behavior		
Never	7189	29.4
Little	11,207	45.8
Sometimes	5476	22.4
Often	565	2.3
Always	38	0.2

**Table 2:** Odds ratios pertaining to poor work-recreation balance conditions for demographic characteristics via multinomial logistic regression modeling.

Independent Variables	Rarely			Sometimes		
	OR	95% CI	*p*-value	OR	95% CI	*p*-value
Age						
≤25	1.44	1.05–1.97	0.024	0.98	0.88–1.10	0.748
>25 and ≤35	2.33	1.68–3.23	＜0.001	1.46	1.29–1.65	＜0.001
>35 and ≤45	1.81	1.29–2.53	0.001	1.27	1.12–1.44	＜0.001
>45	Reference			Reference		
Gender						
Man	1.47	1.30–1.65	＜0.001	1.01	0.96–1.06	0.758
Woman	Reference			Reference		
BMI						
Non-overweight	0.92	0.81–1.04	0.171	0.98	0.93–1.03	0.384
Overweight	Reference			Reference		
Marital status						
Unmarried	0.71	0.63–0.80	＜0.001	0.74	0.71–0.78	＜0.001
Married or others	Reference			Reference		
Education level						
Less than junior high school	3.93	3.30–4.68	＜0.001	1.87	1.70–2.04	＜0.001
High school or college	2.20	1.93–2.51	＜0.001	1.47	1.39–1.56	＜0.001
Bachelor degree or above	Reference			Reference		
Professional						
Professional	1.61	1.36–1.91	＜0.001	1.47	1.37–1.57	＜0.001
Manager	1.70	1.41–2.05	＜0.001	1.51	1.39–1.63	＜0.001
Clerk	2.14	1.22–3.78	0.008	1.65	1.26–2.14	＜0.001
Soldier	0.83	0.20–3.44	0.797	1.77	1.16–2.71	0.008
Production personnel	2.21	1.57–3.12	＜0.001	1.78	1.52–2.08	＜0.001
Operating personnel	4.75	2.66–8.50	＜0.001	1.78	1.23–2.58	0.002
Business, service personnel	3.23	2.70–3.87	＜0.001	1.97	1.80–2.15	＜0.001
Freelancer	3.44	2.42–4.88	＜0.001	1.90	1.57–2.30	＜0.001
No occupation (housewife, *etc.*)	3.65	2.77–4.80	＜0.001	2.12	1.83–2.46	＜0.001
Others	2.18	0.65–7.27	0.205	1.69	0.96–2.98	0.070
High school student	Reference	Reference				
Yes	1.46	1.28–1.66	＜0.001	1.14	1.08–1.21	＜0.001
Never and previously	Reference			Reference		
Drinking behavior						
Yes	1.54	1.32–1.80	＜0.001	1.17	1.09–1.26	＜0.001
Never	Reference			Reference		

Abbreviations: BMI, body mass index; OR, odds ratio; CI, confidence interval.

**Table 3:** The comparison of work-recreation balance frequency in a health-promoting lifestyle and health status (included the factors) using one-way ANOVA.

Dependent Variables	Work-Recreation Balance Frequency						
	Group 1: Rarely	Group 2: Sometimes	Group 3: Always	F Value	*p*-Value	Partial Eta Squared	Multiple Comparisons
	(*n* = 1194)	(*n* = 10,276)	(*n* = 13,005)				
**Health-promoting lifestyle**							
Self-realization	20.47 ± 5.11	22.76 ± 4.41	26.75 ± 4.70	2697.76	＜0.001	0.18	G1 < G2 <G3 **
Health responsibility	14.19 ± 3.55	16.36 ± 3.29	18.80 ± 4.41	1502.56	＜0.001	0.11	G1 < G2 <G3 **
Sports and exercise	12.53 ± 3.90	15.12 ± 3.70	18.25 ± 4.77	2094.56	＜0.001	0.15	G1 < G2 <G3 **
Nutrition	16.95 ± 3.96	19.34 ± 3.61	22.01 ± 4.30	1812.56	＜0.001	0.13	G1 < G2 <G3 **
Interpersonal relationships	19.48 ± 4.13	21.82 ± 3.57	25.77 ± 4.07	3769.73	＜0.001	0.24	G1 < G2 <G3 **
Stress management	15.39 ± 3.21	18.36 ± 2.67	23.00 ± 3.37	8175.16	＜0.001	0.41	G1 < G2 <G3 **
Total score	98.84 ± 16.28	113.79 ± 14.78	134.67 ± 19.18	5239.31	＜0.001	0.31	G1 < G2 <G3 **
**Health status**							
Health (*n* = 4503)	78.63 ± 4.95	78.80 ± 5.14	80.73 ± 5.60	46.82	＜0.001	0.02	G1 < G2 <G3 **
SHS (*n* = 11,337)	60.27 ± 9.41	63.06 ± 7.95	66.90 ± 7.11	450.39	＜0.001	0.07	G1 < G2 <G3 **
Physiological							
Physical symptoms	52.87 ± 16.83	55.69 ± 15.61	59.93 ± 16.24	122.54	＜0.001	0.02	G1 < G2 <G3 **
Organ function	63.96 ± 14.62	64.73 ± 13.26	66.19 ± 13.02	20.06	＜0.001	0.01	G1 < G2 <G3 **
Body movement function	87.40 ± 16.40	87.86 ± 14.47	90.37 ± 13.36	46.96	＜0.001	0.01	G1 < G2 <G3 **
Energy	67.63 ± 22.15	73.19 ± 18.35	78.45 ± 17.16	175.55	＜0.001	0.03	G1 < G2 <G3 **
Psychological							
Positive emotion	55.12 ± 19.05	60.07 ± 15.98	66.43 ± 14.93	299.55	＜0.001	0.05	G1 < G2 <G3 **
Mental symptoms	61.38 ± 15.98	61.97 ± 13.62	64.07 ± 13.05	33.18	＜0.001	0.01	G1 < G2 <G3 **
Cognitive function	47.49 ± 17.32	52.18 ± 14.42	57.09 ± 14.39	223.65	＜0.001	0.04	G1 < G2 <G3 **
Social							
Social adaptation	57.63 ± 16.13	61.49 ± 12.74	66.67 ± 11.65	311.10	＜0.001	0.05	G1 < G2 <G3 **
Social resource	47.27 ± 19.04	53.61 ± 17.09	59.84 ± 17.41	260.74	＜0.001	0.04	G1 < G2 <G3 **
Social support	53.72 ± 18.53	59.16 ± 15.62	63.05 ± 15.64	146.47	＜0.001	0.03	G1 < G2 <G3 **
Disease (*n* = 8635)	56.66 ± 11.04	60.65 ± 9.82	67.95 ± 9.71	707.78	＜0.001	0.14	G1 < G2 <G3 **
Total score	59.20 ± 10.75	63.53 ± 9.85	71.01 ± 9.74	2087.83	＜0.001	0.15	G1 < G2 <G3 **

Data presented as mean ± standard deviation. ANOVA indicates analysis of variance. The Bonferroni method was used for multiple comparisons. ** *p* < 0.001 (significant after Bonferroni correction for *post hoc* analysis).

**Table 4:** Work-recreation balance frequency and health-promoting lifestyle.

Dependent Variables	Work-Recreation Balance Conditions					
	Rarely			Sometimes		
	aOR	95% CI	*p*-value	aOR	95% CI	*p*-value
**Health-promoting lifestyle**						
Poor	388.02	160.03–940.84	＜0.001	132.62	100.41–175.16	＜0.001
General	11.24	4.64–27.20	＜0.001	18.05	13.86–23.50	＜0.001
Good	Reference			Reference		
**Self-realization**						
Poor	36.18	28.87–45.35	＜0.001	12.58	11.21–14.11	＜0.001
General	3.99	3.26–4.88	＜0.001	3.63	3.39–3.88	＜0.001
Good	Reference			Reference		
**Health responsibility**						
Poor	15.99	6.60–38.75	＜0.001	17.88	12.61–25.37	＜0.001
General	2.45	0.96–6.01	0.051	6.03	4.24–8.57	＜0.001
Good	Reference			Reference		
**Sports and exercise**						
Poor	18.88	11.27–31.63	＜0.001	13.76	11.52–16.43	＜0.001
General	2.28	1.33–3.88	0.003	4.62	3.87–5.53	＜0.001
Good	Reference			Reference		
**Nutrition**						
Poor	23.52	14.03–39.44	＜0.001	12.33	10.42–14.59	＜0.001
General	3.26	1.94–5.48	＜0.001	4.52	3.84–5.33	＜0.001
Good	Reference			Reference		
**Interpersonal relationships**						
Poor	125.73	87.75–180.15	＜0.001	27.88	24.21–32.09	＜0.001
General	7.66	5.46–10.76	＜0.001	4.98	4.56–5.43	＜0.001
Good	Reference			Reference		
**Stress management**						
Poor	826.24	499.16–1367.66	＜0.001	253.79	206.13–312.48	＜0.001
General	8.66	5.32–14.11	＜0.001	15.76	13.58–18.29	＜0.001
Good	Reference			Reference		

Abbreviations: aOR, adjusted odds ratio; CI, confidence interval. Demographic variables, including age, gender, BMI, marital status, education level, occupation, unit, drinking behavior, and smoking status were adjusted.

**Table 5:** Adjusted odds ratios (aORs) pertaining to work-recreation balance frequency and health status via multinomial logistic regression modeling.

Variables	Work-Recreation Balance Conditions					
	Rarely			Sometimes		
	aOR	95% CI	*p*-Value	aOR	95% CI	*p*-Value
Health Status	0.08	0.05–0.11	＜0.001	0.26	0.24–0.29	＜0.001
SHS	1.69	1.49–1.92	＜0.001	1.71	1.62–1.81	＜0.001
Physiological	3.78	3.21–4.45	＜0.001	2.39	2.23–2.57	＜0.001
Psychological	5.24	4.35–6.33	＜0.001	3.10	2.89–3.33	＜0.001
Social	6.77	5.46–8.38	＜0.001	3.30	3.07–3.53	＜0.001
Disease Status	1.88	1.65–2.14	＜0.001	1.35	1.27–1.43	＜0.001

Abbreviations: aOR, adjusted odds ratio; CI, confidence interval; SHS, suboptimal health status. Demographic variables, including age, gender, BMI, marital status, education level, occupation, unit, drinking behavior, and smoking status were adjusted.
